# Evaluation of Environmental Safety Concentrations of DMSA Coated Fe_2_O_3_-NPs Using Different Assay Systems in Nematode *Caenorhabditis elegans*

**DOI:** 10.1371/journal.pone.0043729

**Published:** 2012-08-17

**Authors:** Qiuli Wu, Yiping Li, Meng Tang, Dayong Wang

**Affiliations:** 1 Key Laboratory of Environmental Medicine Engineering in Ministry of Education, Department of Biochemistry and Molecular Biology, Medical School of Southeast University, Nanjing, China; 2 School of Public Health, Southeast University, Nanjing, China; Zhejiang University School of Medicine, China

## Abstract

Dimercaptosuccinic acid (DMSA) coating improves the uptake efficiency presumably by engendering the Fe_2_O_3_-NPs. In the present study, we investigated the possible environmental safety concentrations of Fe_2_O_3_-NPs using different assay systems in nematode *Caenorhabditis elegans* with lethality, development, reproduction, locomotion behavior, pharyngeal pumping, defecation, intestinal autofluorescence and reactive oxygen species (ROS) production as the endpoints. After exposure from L4-larvae for 24-hr, DMSA coated Fe_2_O_3_-NPs at concentrations more than 50 mg/L exhibited adverse effects on nematodes. After exposure from L1-larvae to adult, DMSA coated Fe_2_O_3_-NPs at concentrations more than 500 μg/L had adverse effects on nematodes. After exposure from L1-larvae to day-8 adult, DMSA coated Fe_2_O_3_-NPs at concentrations more than 100 μg/L resulted in the adverse effects on nematodes. Accompanied with the alterations of locomotion behaviors, ROS production was pronouncedly induced by exposure to DMSA coated Fe_2_O_3_-NPs in the examined three assay systems, and the close associations of ROS production with lethality, growth, reproduction, locomotion behavior, pharyngeal pumping, defecation, or intestinal autofluorescence in nematodes exposed to DMSA coated Fe_2_O_3_-NPs were confirmed by the linear regression analysis. Moreover, mutations of *sod-2* and *sod-3* genes, encoding Mn-SODs, showed more susceptible properties than wild-type when they were used for assessing the DMSA coated Fe_2_O_3_-NPs-induced toxicity, and the safety concentrations for DMSA coated Fe_2_O_3_-NPs should be defined as concentrations lower than 10 μg/L in *sod-2* and *sod-3* mutant nematodes.

## Introduction

Iron oxide nanoparticles (NPs) hold immense potential in a vast variety of applications such as magnetic resonance imaging, targeted delivery of drugs or genes, tissue engineering, targeted destruction of tumor, magnetic transfections, chelation therapy and environmental catalysis [1]–[2]. Although, the potential benefits of Fe-NPs are considerable, exposure to Fe-NPs can result in the significant cytotoxicity such as inflammation, formation of apoptosis, impaired mitochondrial function, membrane leakage, oxidative stress, damage on cell function, DNA damage, chromosomal damage and chromosome condensation [2]–[4]. Besides the cytocotocity, the *in vivo* toxicity assays further indicated that Fe-NPs exposure caused lung inflammation associated with increased cytokine productions in lymph node cell cultures and decreased pulmonary immune response against sleep erythrocytes, disrupted embryo development, and induced oxidative injury in peritoneal macrophage in mice [5]–[7]. Fe-NPs exposure also resulted in the potential lung and systemic cumulative toxicity, and histopathological alterations of liver and spleen in rats [8]–[9]. Moreover, the ecotoxic investigations demonstrated that Fe-NPs exposure induced the lipid peroxidation in excised mussel gills, and caused the oxidative stress, disturbance of antioxidative balance, and some histopathological and morphological alterations in medaka [10]–[11]. The increased production of novel food additives in the form of Fe-NPs bring attention to the potential environmental health risks [12], and the recent increasing interest in the use of Fe-NPs for wastewater treatment [11] may further bring about the amplification of the possible environmental risk. However, present knowledge concerning the ecotoxic effects of Fe-NPs is very limited and merits to be documented more fully.

The model animal of nematode *Caenorhabditis elegans*, a free-living nematode with the abundance in soil ecosystems, is useful for environmental and toxicological studies of toxicants from whole-animal level down to single cell level [13]. *C. elegans* can be explored to assess the toxicities of both contaminated soil and contaminated river water or sediments [14]–[17]. Endpoints of lethality, development, reproduction, locomotion behavior, lifespan, pharyngeal pumping, defecation, intestinal autofluorescence, stress response, and oxidative stress can be used to evaluate the acute or chronic toxicity of environmental toxicants [18]–[31]. So far, *C. elegans* has been successfully used for the toxicity evaluation of different nano-materials, such as Al-NPs, Ti-NPs, Ce-NPs, Zn-NPs, Ag-NPs, Si-NPs, fullerene, and quantum dots [32]–[45]. It has been proven that *C. elegans* can be used to evaluate the toxicity of specific toxicants at environmental relevant concentrations [37], [39]. The mean lifespan of nematodes was significantly decreased even at the exposure level of 1 nM for CeO_2_-NPs [39]. The locomotion behavior, intestinal autofluorescence and reactive oxygen species (ROS) production were significantly altered in nematodes chronically exposed to 13 μg/L of Cr(VI) [46]. Nevertheless, no report was raised so far to investigate the ecotoxic effects of Fe-NPs using *C. elegans* as a toxicity assay system.

For uncoated Fe-NPs, it has been shown that they are NPs-specific cytotoxic [47]. To effectively use Fe-NPs in the medical treatment or magnetic resonance imaging, various organic coatings have been employed as a means of optimizing the delivery of Fe-NPs to or into the cells [48]–[49]. A simple dimercaptosuccinic acid (DMSA) coating can improve the uptake efficiency presumably by engendering the Fe-NPs with an anionic charge, resulting in nonspecific adsorption to the cell surface followed by endocytosis into the cells [50]. In the present study, we investigated the possible environmental safety concentrations of Fe_2_O_3_-NPs using different assay systems in nematode *Caenorhabditis elegans*.

## Results

### Toxicity evaluation in nematodes exposed to DMSA coated Fe_2_O_3_-NPs at the L4-larvae stage for 24-hr

**Figure 1:**
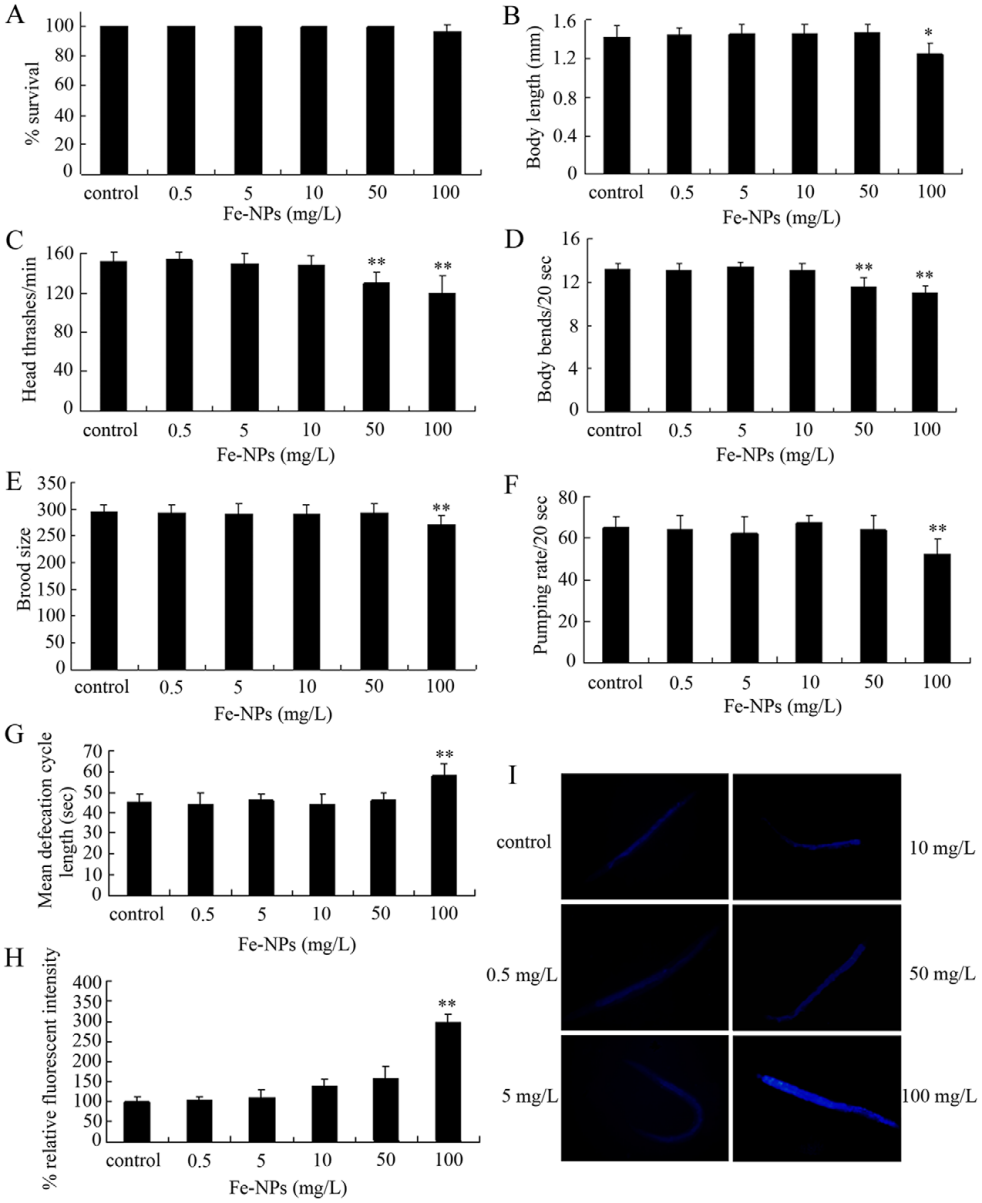
Toxicity evaluation in nematodes exposed to DMSA coated Fe_2_O_3_-nanoparticles at the L4-larvae stage for 24-hr. (A) Comparison of lethality in nematodes exposed to different concentrations of Fe_2_O_3_-nanoparticles. (B) Comparison of body length in nematodes exposed to different concentrations of Fe_2_O_3_-nanoparticles. (C) Comparison of head thrash in nematodes exposed to different concentrations of Fe_2_O_3_-nanoparticles. (D) Comparison of body bend in nematodes exposed to different concentrations of Fe_2_O_3_-nanoparticles. (E) Comparison of brood size in nematodes exposed to different concentrations of Fe_2_O_3_-nanoparticles. (F) Comparison of pumping rate in nematodes exposed to different concentrations of Fe_2_O_3_-nanoparticles. (G) Comparison of mean defecation cycle length in nematodes exposed to different concentrations of Fe_2_O_3_-nanoparticles. (H) Comparison of intestinal autofluorescence in nematodes exposed to different concentrations of Fe_2_O_3_-nanoparticles. (I) Pictures showing the intestinal autofluorescence in nematodes exposed to different concentrations of Fe_2_O_3_-nanoparticles. Bars represent mean ± S.E.M. * *p*<0.05, ** *p*<0.01.

We first investigated the possible adverse effects of DMSA coated Fe_2_O_3_-NPs exposure at the L4-larvae for 24-hr on nematodes. Exposure to 0.5–100 mg/L of DMSA coated Fe_2_O_3_-NPs at the L4-larvae for 24-hr did not obviously influence the survival of nematodes (Fig. 1A). Similarly, exposure to 0.5–50 mg/L of DMSA coated Fe_2_O_3_-NPs did not significantly affect the body length of nematodes (Fig. 1B), exposure to 0.5–10 mg/L of DMSA coated Fe_2_O_3_-NPs did not noticeably influence the locomotion behavior as indicated by head thrash and body bend in nematodes (Fig. 1C and 1D), exposure to 0.5–50 mg/L of DMSA coated Fe_2_O_3_-NPs did not change the brood size of nematodes (Fig. 1E), exposure to 0.5–50 mg/L of DMSA coated Fe_2_O_3_-NPs did not obviously alter the pumping rate and defecation (Fig. 1F and 1G), and exposure to 0.5–50 mg/L of DMSA coated Fe_2_O_3_-NPs did not significantly induce the intestinal autofluorescence of nematodes (Fig. 1H and 1I). In contrast, exposure to 100 mg/L of DMSA coated Fe_2_O_3_-NPs significantly reduced the body length and brood size (Fig. 1B and 1C), decreased the pumping rate (Fig. 1F), increased the mean defecation cycle length (Fig. 1G), and induced the intestinal autolfuorescence of nematodes (Fig. 1H and 1I). Especially, exposure to 50–100 mg/L of DMSA coated Fe_2_O_3_-NPs significantly decreased both the head thrashes and the body bends in nematodes (Fig. 1C and 1D). Therefore, acute exposure to 50–100 mg/L of DMSA coated Fe_2_O_3_-NPs may exhibit adverse effects on nematodes.

### Toxicity evaluation in nematodes exposed to DMSA coated Fe_2_O_3_-NPs from L1-larvae to adult

**Figure 2:**
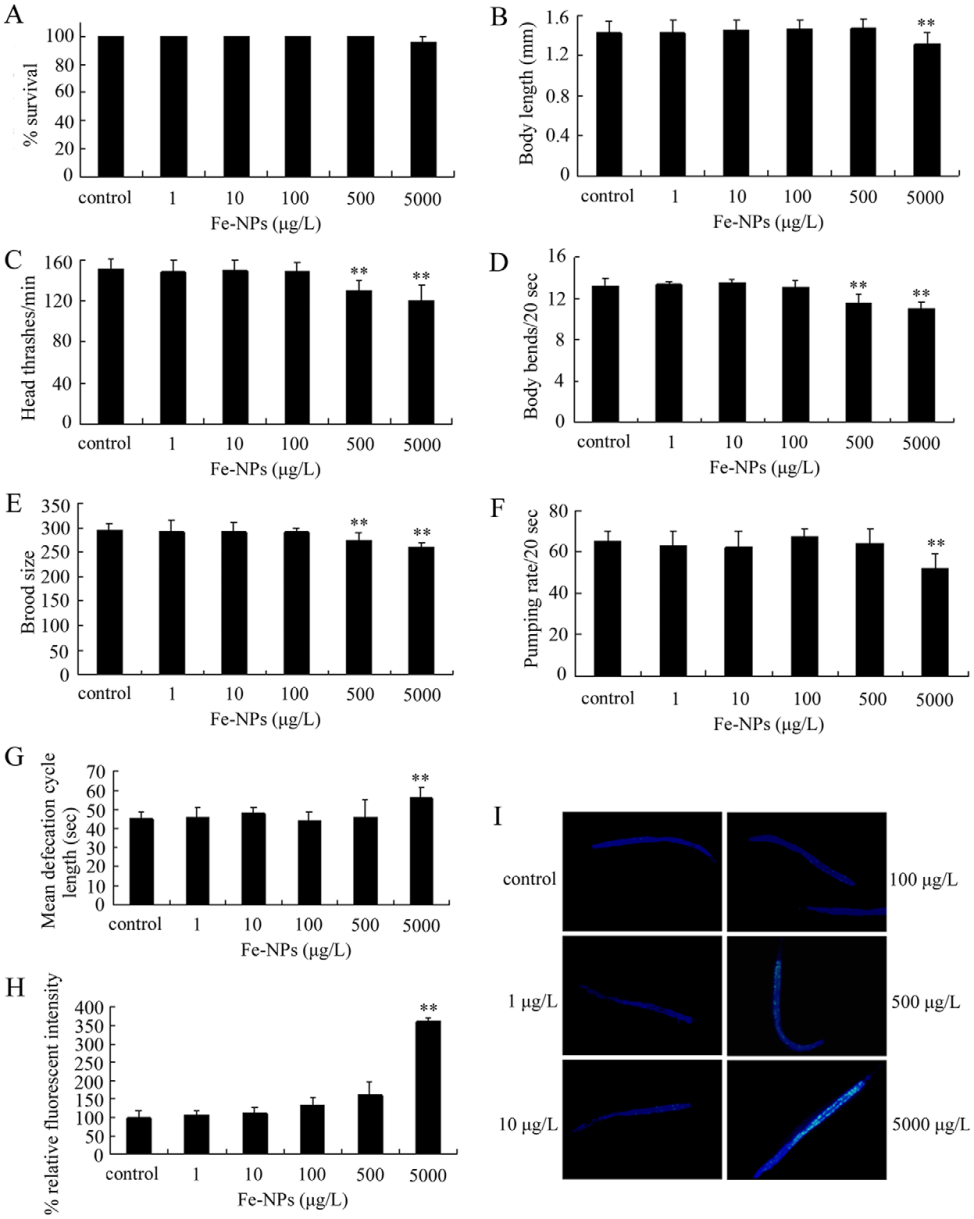
Toxicity evaluation in nematodes exposed to DMSA coated Fe_2_O_3_-nanoparticles from L1-larvae to adult. (A) Comparison of lethality in nematodes exposed to different concentrations of Fe_2_O_3_-nanoparticles. (B) Comparison of body length in nematodes exposed to different concentrations of Fe_2_O_3_-nanoparticles. (C) Comparison of head thrash in nematodes exposed to different concentrations of Fe_2_O_3_-nanoparticles. (D) Comparison of body bend in nematodes exposed to different concentrations of Fe_2_O_3_-nanoparticles. (E) Comparison of brood size in nematodes exposed to different concentrations of Fe_2_O_3_-nanoparticles. (F) Comparison of pumping rate in nematodes exposed to different concentrations of Fe_2_O_3_-nanoparticles. (G) Comparison of mean defecation cycle length in nematodes exposed to different concentrations of Fe_2_O_3_-nanoparticles. (H) Comparison of intestinal autofluorescence in nematodes exposed to different concentrations of Fe_2_O_3_-nanoparticles. (I) Pictures showing the intestinal autofluorescence in nematodes exposed to different concentrations of Fe_2_O_3_-nanoparticles. Bars represent mean ± S.E.M. * *p*<0.05, ** *p*<0.01.

Previous studies indicated that exposure to environmental relevant concentrations of CeO_2_-NPs or Al_2_O_3_-NPs from L1-larvae to adult caused the adverse effects in nematodes, implying the sensitivity of L1-larvae to environmental toxicants [39], [43]. We next investigated the possible adverse effects of exposure to DMSA coated Fe_2_O_3_-NPs from L1-larvae to day 1 adult nematodes (approximately 3 days). As shown in Fig. 2, exposure to 1–5000 μg/L of DMSA coated Fe_2_O_3_-NPs did not obviously influence the survival of nematodes. Similarly, exposure to 1–500 μg/L of DMSA coated Fe_2_O_3_-NPs did not affect the body length, pumping rate, and defecation in nematodes, exposure to 1–100 μg/L of DMSA coated Fe_2_O_3_-NPs did not noticeably alter the head thrash, body bend, and brood size in nematodes, and exposure to 1–500 μg/L of DMSA coated Fe_2_O_3_-NPs also did not induce the significant intestinal autofluorescence in nematodes. In contrast, exposure to 5000 μg/L of DMSA coated Fe_2_O_3_-NPs significantly reduced the body length, decreased the pumping rate, increased the mean defecation cycle length, and induced the intestinal autofluorescence. Especially, exposure to 500–1000 μg/L of DMSA coated Fe_2_O_3_-NPs significantly decreased both the locomotion behavior and the brood size of nematodes. These data imply that, after exposure from L1-larvae to adult, DMSA coated Fe_2_O_3_-NPs with concentrations more than 500 μg/L may have adverse effects on nematodes.

### Toxicity evaluation in nematodes exposed to DMSA coated Fe_2_O_3_-NPs from L1-larvae to day-8 adult

**Figure 3:**
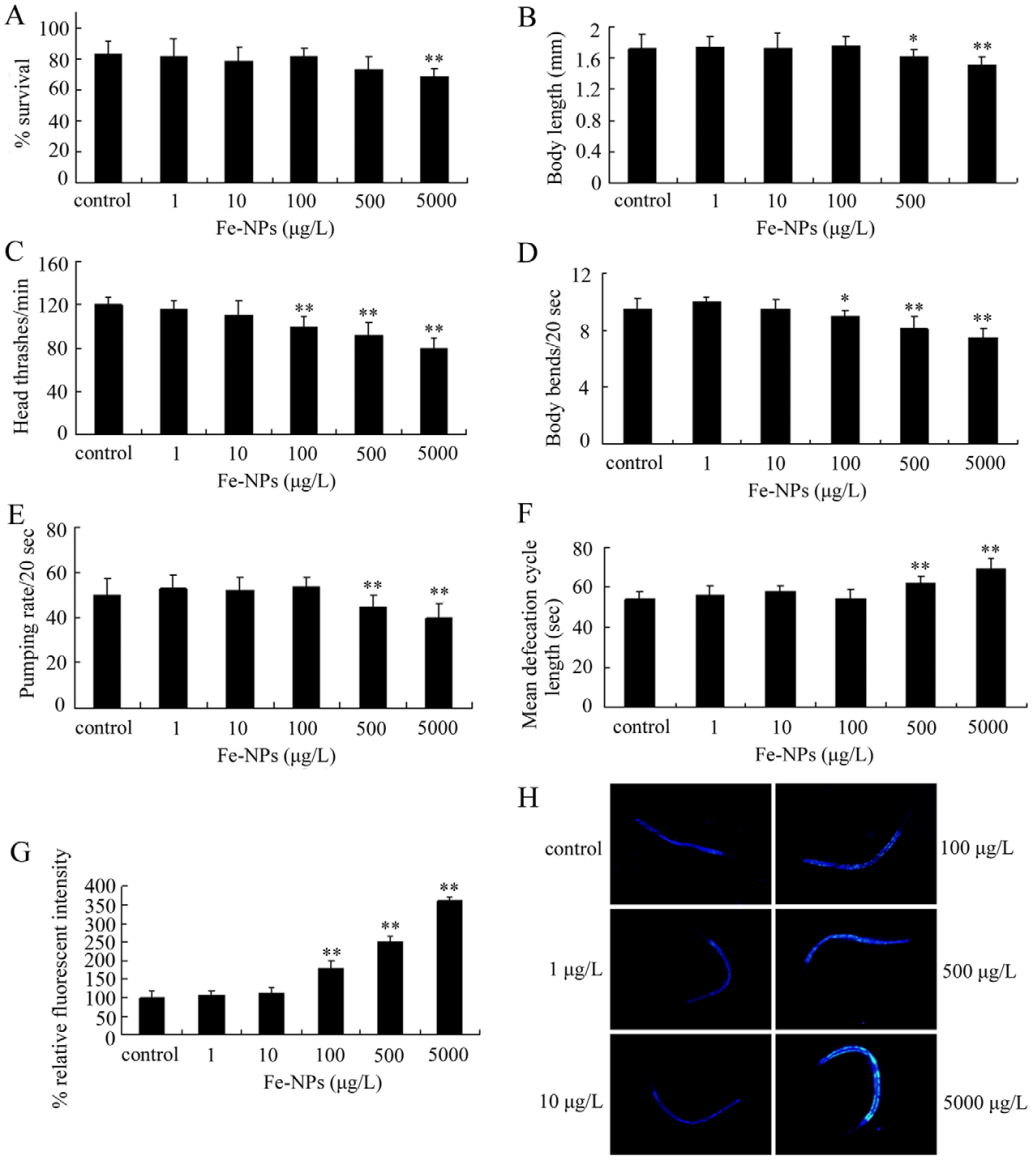
Toxicity evaluation in nematodes exposed to DMSA coated Fe2O3-nanoparticles from L1-larvae to day-8 adult. (A) Comparison of lethality in nematodes exposed to different concentrations of Fe_2_O_3_-nanoparticles. (B) Comparison of body length in nematodes exposed to different concentrations of Fe_2_O_3_-nanoparticles. (C) Comparison of head thrash in nematodes exposed to different concentrations of Fe_2_O_3_-nanoparticles. (D) Comparison of body bend in nematodes exposed to different concentrations of Fe_2_O_3_-nanoparticles. (E) Comparison of pumping rate in nematodes exposed to different concentrations of Fe_2_O_3_-nanoparticles. (F) Comparison of mean defecation cycle length in nematodes exposed to different concentrations of Fe_2_O_3_-nanoparticles. (G) Comparison of intestinal autofluorescence in nematodes exposed to different concentrations of Fe_2_O_3_-nanoparticles. (H) Pictures showing the intestinal autofluorescence in nematodes exposed to different concentrations of Fe_2_O_3_-nanoparticles. Bars represent mean ± S.E.M. * *p*<0.05, ** *p*<0.01.

*C. elegans* can be used for chronic toxicity evaluation [38], [42], [46], [51]. To combine the sensitive value of L1-larvae for toxicity evaluation with the previous chronic assay system in *C. elegans*, we further investigated the possible adverse effects of exposure to DMSA coated Fe_2_O_3_-NPs from L1-larvae to day-8 adult on nematodes. As shown in Fig. 3, different from the toxicity evaluation above, we observed that exposure to 5000 μg/L of DMSA coated Fe_2_O_3_-NPs moderately but significantly decreased the survival of nematodes. Moreover, exposure to 500–5000 μg/L of DMSA coated Fe_2_O_3_-NPs significantly reduced the body length, decreased the pumping rate, prolonged the mean defecation cycle length, and induced the intestinal autofluorescence. Especially, exposure to 100–5000 μg/L of DMSA coated Fe_2_O_3_-NPs significantly decreased the locomotion behaviors and induced the intestinal autofluorescence of nematodes. Therefore, our data suggest that, after exposure from L1-larvae to day-8 adult, DMSA coated Fe_2_O_3_-NPs with concentrations more than 100 μg/L may exhibit adverse effects on nematodes.

### ROS production in nematodes exposed to DMSA coated Fe_2_O_3_-NPs in different assay systems

**Figure 4:**
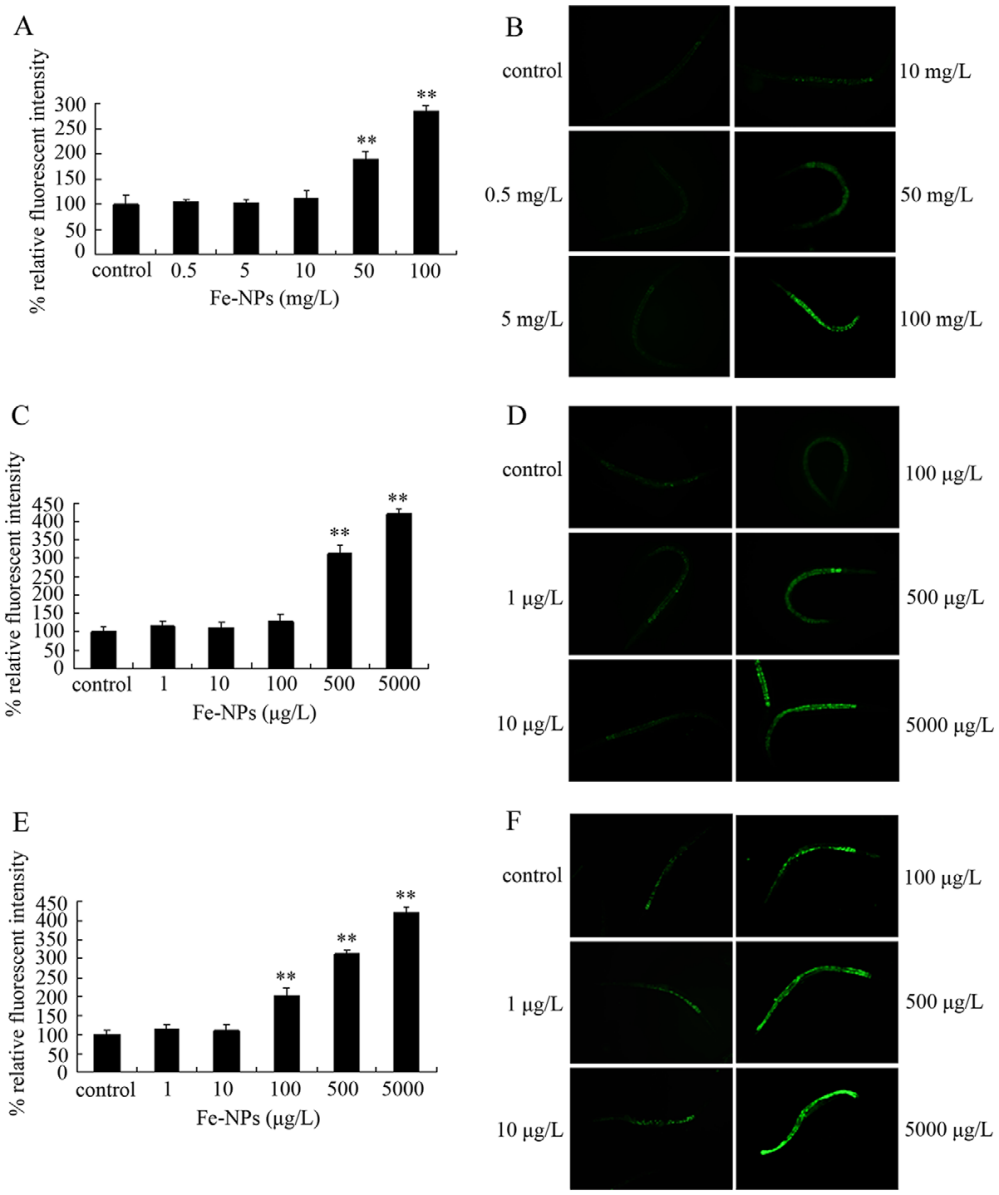
ROS production in nematodes exposed to DMSA coated Fe_2_O_3_-nanoparticles. (A) Comparison of ROS production in nematodes exposed to Fe_2_O_3_-nanoparticles at the L4-larvae stage for 24-hr. (B) Pictures showing the ROS production in nematodes exposed to Fe_2_O_3_-nanoparticles at the L4-larvae stage for 24-hr. (C) Comparison of ROS production in nematodes exposed to Fe_2_O_3_-nanoparticles from L1-larvae to adult. (D) Pictures showing the ROS production in nematodes exposed to Fe_2_O_3_-nanoparticles from L1-larvae to adult. (E) Comparison of ROS production in nematodes exposed to Fe_2_O_3_-nanoparticles from L1-larvae to day-8 adult. (F) Pictures showing the ROS production in nematodes exposed to Fe_2_O_3_-nanoparticles from L1-larvae to day-8 adult. Bars represent mean ± S.E.M. **p<0.01.

Considering the fact that the uncoated Fe_2_O_3_-NPs can induce the oxidative stress [4]–[5], [10]–[11], we examined whether exposure to DMSA coated Fe_2_O_3_-NPs will induce the oxidative stress by analyzing the ROS production in nematodes. After exposure from L4-larvae for 24-hr, 50–100 mg/L of DMSA coated Fe_2_O_3_-NPs induced the significant ROS production in nematodes (Fig. 4A and 4B). After exposure from L1-larvae to adult, 500–5000 μg/L of DMSA coated Fe_2_O_3_-NPs induced the significant ROS production in nematodes (Fig. 4C and 4D). Moreover, after exposure from L1-larvae to day-8 adult, we found that 100–5000 μg/L of DMSA coated Fe_2_O_3_-NPs induced the significant ROS production in nematodes (Fig. 4E and 4F).

To examine the possible associations of ROS production with lethality, growth, reproduction, locomotion behavior, pharyngeal pumping, defecation, or intestinal autolfuorescence in nematodes exposed to different concentrations of DMSA coated Fe_2_O_3_-NPs, the linear regression analysis was further performed. With growth, reproduction, locomotion behavior, pharyngeal pumping, defecation, and intestinal autolfuorescence as the dependent variables and with ROS production as the independent variable, after DMSA coated Fe_2_O_3_-NPs exposure from L4-larvae for 24-hr, ROS production was significantly correlated with growth (R^2^ = 0.856, *p*<0.01), reproduction (R^2^ = 0.913, *p*<0.01), body bend (R^2^ = 0.730, *p*<0.05), head thrash (R^2^ = 0.795, *p*<0.05), pumping rate (R^2^ = 0.861, *p*<0.01), mean defecation cycle length (R^2^ = 0.954, *p*<0.01), and intestinal autofluorescence (R^2^ = 0.977, *p*<0.01) (Table S1). With growth, reproduction, locomotion behavior, pharyngeal pumping, defecation, and intestinal autolfuorescence as the dependent variables and with ROS production as the independent variable, after DMSA coated Fe_2_O_3_-NPs exposure from L1-larvae to adult, ROS production was significantly correlated with growth (R^2^ = 0.688, *p*<0.05), reproduction (R^2^ = 0.881, *p*<0.01), body bend (R^2^ = 0.739, *p*<0.05), head thrash (R^2^ = 0.842, *p*<0.05), pumping rate (R^2^ = 0.863, *p*<0.01), mean defecation cycle length (R^2^ = 0.884, *p*<0.01), and intestinal autofluorescence (R^2^ = 0.993, *p*<0.01) (Table S1). With lethality, growth, locomotion behavior, pharyngeal pumping, defecation, and intestinal autolfuorescence as the dependent variables and with ROS production as the independent variable, after DMSA coated Fe_2_O_3_-NPs exposure from L1-larvae to day-8 adult, ROS production was significantly correlated with lethality (R^2^ = 0.920, *p*<0.01), growth (R^2^ = 0.883, *p*<0.01), body bend (R^2^ = 0.947, *p*<0.01), head thrash (R^2^ = 0.920, *p*<0.01), pumping rate (R^2^ = 0.828, *p*<0.05), mean defecation cycle length (R^2^ = 0.842, *P*<0.05), and intestinal autofluorescence (R^2^ = 0.781, *p*<0.05) (Table S1). Therefore, ROS production was significantly correlated with lethality, growth, reproduction, locomotion behavior, pharyngeal pumping, defecation, or intestinal autofluorescence in nematodes exposed to DMSA coated Fe_2_O_3_-NPs in different toxicity evaluation assay systems.

### Effects of *sod-2* and *sod-3* mutations on locomotion behavior and ROS production in nematodes exposed to DMSA coated Fe_2_O_3_-NPs

**Figure 5:**
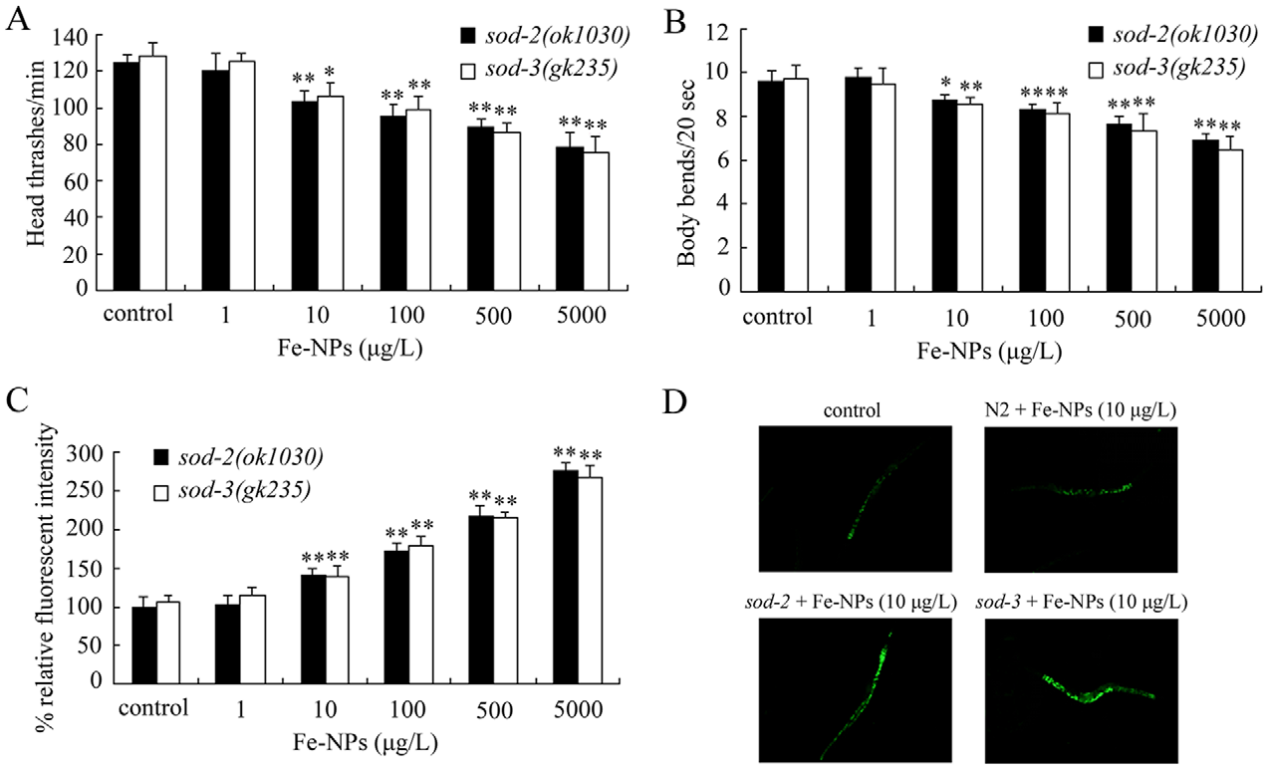
Effects of *sod-2* and *sod-3* mutations on locomotion behavior and ROS production in nematodes exposed to DMSA coated Fe_2_O_3_-nanoparticles from L1-larvae to day-8 adult. (A) Effects of *sod-2* and *sod-3* mutations on head thrashes of nematodes exposed to DMSA coated Fe_2_O_3_-nanoparticles. (B) Effects of *sod-2* and *sod-3* mutations on body bends of nematodes exposed to DMSA coated Fe_2_O_3_-nanoparticles. (C) Effects of *sod-2* and *sod-3* mutations on ROS production in nematodes exposed to DMSA coated Fe_2_O_3_-nanoparticles. (D) Pictures showing the ROS production in nematodes exposed to Fe_2_O_3_-nanoparticles. Bars represent mean ± S.E.M. *p<0.05, **p<0.01.

Previous studies indicated that mutations of some genes, such as *sod-2* and *sod-3* genes encoding Mn-SODs, exhibited more susceptible properties than wild-type nematodes for assessing the nanotoxicity [35], [42]. We further investigated the possible susceptible properties of *sod-2* and *sod-3* mutants to toxicity of DMSA coated Fe_2_O_3_-NPs. Based on the analysis above, we selected two relative sensitive endpoints, locomotion behavior and ROS production, for the further toxicity assay. In *sod-2* and *sod-3* mutants exposed to DMSA coated Fe_2_O_3_-NPs from L1-larvae to day-8 adult, 10–5000 μg/L of DMSA coated Fe_2_O_3_-NPs significantly inhibited both the head thrashes and the body bends of nematodes (Fig. 5A and 5B). Further, the ROS productions were significantly induced in *sod-2* and *sod-3* mutants exposed to 10–5000 μg/L of DMSA coated Fe_2_O_3_-NPs from L1-larvae to day-8 adult (Fig. 5C and 5D). More interestingly, we found that double mutations of *sod-2* and *sod-3* gens resulted in the more significant (*p*<0.01) decreases of head thrash and body bend, and increases of ROS production in nematodes exposed to 10 μg/L of DMSA coated Fe_2_O_3_-NPs from L1-larvae to day-8 adult compared with the adverse effects from single mutations of *sod-2* or *sod-3* gens (Fig. S1).

## Discussion

Surface-coated Fe_2_O_3_-NPs has been proposed for medical treatment or as a contrast agent in magnetic resonance imaging. The *in vitro* toxicity assay on Fe_2_O_3_-NPs has shown the low toxicity and no clear difference between different particle sizes [4]. Enhanced endocytosis by DMSA coating is an efficient method for intracellular delivery of Fe_2_O_3_-NPs. Previous study has shown little or no *in vivo* toxicity for DMSA [52]. In the current study, with lethality, growth, reproduction, locomotion behavior, pharyngeal pumping, defecation, and intestinal autofluorescence as the endpoints, we investigated the adverse effects of exposure to DMSA coated Fe_2_O_3_-NPs for 24-hr on nematodes, and found that exposure to 50–100 mg/L of DMSA coated Fe_2_O_3_-NPs had adverse effects on nematodes (Fig. 1). Although exposure to DMSA coated Fe_2_O_3_-NPs at the examined concentrations did not obviously influence the survival of nematodes, exposure to 100 mg/L of DMSA coated Fe_2_O_3_-NPs altered the growth, reproduction, locomotion behavior, pharyngeal pumping, defecation, and intestinal autofluorescence (Fig. 1). Especially, exposure to 50 mg/L of DMSA coated Fe_2_O_3_-NPs decreased the locomotion behavior of nematodes, implying the locomotion behavior is the most sensitive one among the used endpoints for assessing the toxicity of DMSA coated Fe_2_O_3_-NPs (Fig. 1). Therefore, after exposure from L4-larvae for 24-hr, only relatively high concentrations of DMSA coated Fe_2_O_3_-NPs can cause the adverse effects on nematodes. Previous *in vitro* toxicity assay on DMSA coated Fe_2_O_3_-NPs (5–12 nm) also indicated that exposure at concentrations of 1.5–15 mM resulted in a diminishing viability and capacity of PC12 cells to extend neuritis in response to their putative biological cue, i.e. nerve growth factor [3].

To evaluate the possible environmental safety concentrations for DMSA coated Fe_2_O_3_-NPs, we further explored two other toxicity assay systems. With lethality, growth, reproduction, locomotion behavior, pharyngeal pumping, defecation, and intestinal autofluorescence as the endpoints, we investigated the adverse effects of exposure to DMSA coated Fe_2_O_3_-NPs from L1-larvae to adult on nematodes and found that DMSA coated Fe_2_O_3_-NPs with concentrations more than 500 μg/L exhibited adverse effects on nematodes (Fig. 2). Exposure to DMSA coated Fe_2_O_3_-NPs from L1-larvae to adult at the examined concentrations also did not obviously affect the survival of nematodes; however, exposure to 5000 μg/L of DMSA coated Fe_2_O_3_-NPs altered the growth, reproduction, locomotion behavior, pharyngeal pumping, defecation, and intestinal autofluorescence and exposure to 500 μg/L of DMSA coated Fe_2_O_3_-NPs further suppressed the locomotion behavior of nematodes (Fig. 2). In contrast to these, with lethality, growth, locomotion behavior, pharyngeal pumping, defecation, and intestinal autofluorescence as the endpoints, we examined the adverse effects of exposure to DMSA coated Fe_2_O_3_-NPs from L1-larvae to day-8 adult on nematodes, and found that DMSA coated Fe_2_O_3_-NPs with concentrations more than 100 μg/L resulted in the toxic effects on nematodes (Fig. 3). Different from the observations in other assay systems, exposure to 5000 μg/L of DMSA coated Fe_2_O_3_-NPs from L1-larvae to day-8 adult reduced the survival rate of nematodes (Fig. 3). Moreover, exposure to 100 μg/L of DMSA coated Fe_2_O_3_-NPs from L1-larvae to day-8 adult decreased the locomotion behavior and induced the significant intestinal autofluorescence (Fig. 3). Among the used assay systems, the assay system of exposure from L1-larvae to day-8 adult combines both the value of assay system of exposure from L1-larvae to adult [39], [42] and the value of assay system of exposure from day-1 adult to day-8 adult [46]. With the aid of the above toxicity assay systems, we showed here that DMSA coated Fe_2_O_3_-NPs with concentrations lower than 100 μg/L should be relative safe in the environment. Nevertheless, different from some other NPs [53]–[54], so far we still do not know the exact environmental concentrations or predicated environmental concentrations of Fe_2_O_3_-NPs.

The environmental safety concentrations for Fe_2_O_3_-NPs revealed in this study are somewhat different from previous publications. In excised mussel gills, exposure to 1000 μg/L of Fe_2_O_3_-NPs (50 nm) induced the lipid peroxidation and impairment on lysosomal stability in circulating blood cells [11]. In addition, exposure to 500 μg/L of Fe-NPs (30 nm) with a biodegradable polymer modification caused the decrease of superoxide dismutase in medaka [10]. At least three possibilities will explain these differences. One possibility is that the size of Fe_2_O_3_-NPs used in this study is different from those used in previous studies [10]–[11]. Another possibility is that the assay system used in this study is different from those used in other organisms [10]–[11]. In addition, we still can not exclude such a possibility that *C. elegans* may be more sensitive than cell line or medaka while assessing the nanotoxicity, as implied in the studies on the Al_2_O_3_-NPs toxicity in *C. elegans* [42]–[43].

In *C. elegans*, *sod-2* and *sod-3* mutants are very sensitive to the oxidative stress [55]. Furthermore, our data here suggest that *sod-2* and *sod-3* mutants were more susceptible than wild-type to DMSA coated Fe_2_O_3_-NPs exposure-induced toxicity (Fig. 5). In *sod-2* and *sod-3* gene mutation backgrounds, the relative safety concentrations for DMSA coated Fe_2_O_3_-NPs should be defined as concentrations lower than 10 μg/L in the environment. Therefore, on the one hand, *sod-2* and *sod-3* mutants can be used to detect the possible potential toxicity from DMSA coated Fe_2_O_3_-NPs; on the other hand, under the specific sensitive mutation or physiological backgrounds, the environmental safety concentrations for specific nanomaterials should be carefully investigated and defined. More interestingly, the relative safety concentrations for DMSA coated Fe_2_O_3_-NPs in *sod-2; sod-3* double mutants should be defined as concentrations even lower than those in *sod-2* or *sod-3* single mutants in the environment (Fig. S1).

Previous studies indicated that exposure to uncoated Fe_2_O_3_-NPs (30 nm) caused more H_2_O_2_, OH and O_2_^−^ free radicals in peritoneal macrophage of mice compared with control, exposure to uncoated Fe_2_O_3_-NPs (29 nm) induced oxidative DNA lesions in human cell line A549, exposure to uncoated Fe_2_O_3_-NPs (50 nm) resulted in the lipid peroxidation in excised mussel gills, and exposure to Fe-NPs (30 nm) with a biodegradable polymer modification induced the decrease of superoxide dismutase, increase of malondialdehyde, and reduced glutathione in medaka [4]–[5], [10]–[11]. In the present study, our data further demonstrated that, accompanied with the alterations of locomotion behaviors induced by exposure to DMSA coated Fe_2_O_3_-NPs, ROS production was pronouncedly induced by exposure to DMSA coated Fe_2_O_3_-NPs in the used three assay systems (Fig. 4). Moreover, the linear regression analysis confirmed the close association of the observed ROS production with the altered lethality, growth, reproduction, locomotion behavior, pharyngeal pumping, defecation, or intestinal autofluorescence in nematodes exposed to DMSA coated Fe_2_O_3_-NPs in different toxicity evaluation assay systems (Table S1). These data also imply that ROS production is also a very sensitive endpoint used for environmental safety assessment of specific nanomaterials.

In summary, we examined the possible environmental safety concentrations for DMSA-coated Fe_2_O_3_-NPs using three different toxicity assay systems in nematodes, and indicated that DMSA coated Fe_2_O_3_-NPs with concentrations lower than 100 μg/L should be relative safe in the environment. In contrast, in *sod-2* and *sod-3* mutants, the environmental safety concentrations for DMSA coated Fe_2_O_3_-NPs should be defined as concentrations lower than 10 μg/L. Moreover, we provide some evidence to prove that the toxicity induced by DMSA coated Fe_2_O_3_-NPs is at least partially due to the formation of oxidative stress in nematodes.

## Materials and Methods

### Reagents and preparation of DMSA coated Fe_2_O_3_-NPs suspensions

**Figure 6:**
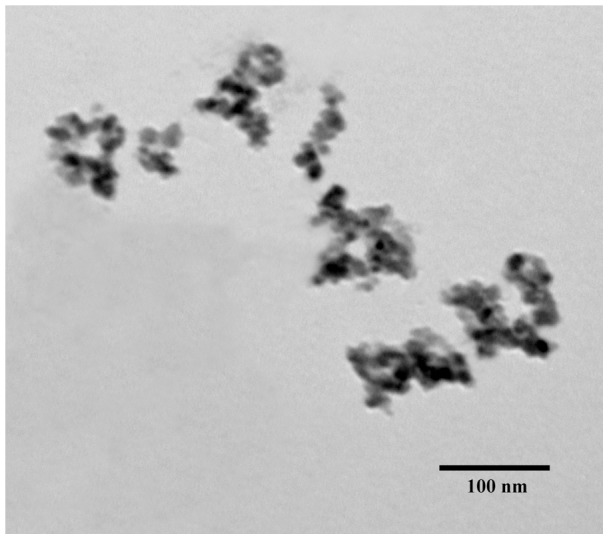
TEM image of 9 nm DMSA coated Fe_2_O_3_-nanoparticles.

DMSA coated Fe_2_O_3_-NPs was the gift from Prof. Yu Zhang [56]. The size of particles was 9 nm as measured by transmission electron microscopy (TEM, JEM 200 CX) (Fig. 6). We determined the crystal structure by powder X-ray diffraction (XRD, Rigaku, D/Max-RA, λ = 1.5405×10^−10^ m, Cu*K*), measured the specific surface area by N2 sorption at 77 K using a NOVA 1000e instrument, tested the particle size distribution in K medium by dynamic light scattering (DLS) measurement (Brookhaven Instruments Corp., Holsvile, NY), and analyzed the zeta potential for Fe_2_O_3_-NPs with a Nano Zetasizer (Malvern Instrument Ltd., Malvern, UK). The detailed properties of Fe_2_O_3_-NPs are shown in Table S2. The prepared stock suspension concentrations of Fe_2_O_3_-NPs were 0.5, 5, 10, 50, and 100 mg/L, and 1, 10, 100, 500, and 5000 μg/L. Series of stock suspensions of Fe_2_O_3_-NPs were prepared in a K-medium [57], and dispersed for 20 min by probe sonication at 100 W and 40 kHz for 30 min to form the used suspensions. All the other chemicals were obtained from Sigma-Aldrich (St. Louis, MO, USA).

### Strain preparation

Nematodes used were wild-type N2, RB1072 [*sod-2(ok1030)*], VC433 [*sod-3(gk235)*], which were maintained on nematode growth medium (NGM) plates seeded with *Escherichia coli* OP50 at 20°C [58]. Gravid nematodes were washed off the plates into centrifuge tubes, and were lysed with a bleaching mixture (0.45 M NaOH, 2% HOCl). Age synchronous populations of L1-larvae or L4 larvae nematodes were obtained by the collection as described [59]. The adult were washed with K medium (50 mM NaCl, 30 mM KCl, 10 mM NaOAc, pH 5.5) [57].

Exposures to different concentrations of Fe_2_O_3_-NPs were performed in three assay systems: (1) from the L4-larvae stage in K medium of 12-well sterile tissue culture plates for 24-hr, (2) from the L1-larvae to day 1 adult in K medium of 12-well sterile tissue culture plates, and (3) from L1-larvae to day-8 adult in K medium of 12-well sterile tissue culture plates (chronic exposure). All the exposures were performed at 20°C incubator in the presence of food. Five hundred microliters of exposure suspension solution was added into each well of the 12-well sterile tissue culture plates. The exposure suspension solutions were freshly prepared prior to use. For the chronic exposure assay, 5′-fluoro-2′-deoxyuridine (FUdR), an inhibitor of DNA synthesis, was used to prevent production of offspring from reproducing without otherwise interfering with the organism's post-maturational development [60] by adding at a final concentration of 25 μM when the examined nematodes developed into the L4-larvae stage. For chronic exposure, the exposed nematodes were transferred from day 1 to day 8 to a new well with Fe_2_O_3_-NPs suspension solution with food each day in order to ensure the food supply.

### Lethality, growth, reproduction, metabolism, and locomotion behavior

We used the percentage of survival animals to evaluate lethality of nematodes. Following exposure, inactive ones were scored under a dissecting microscopy and nematodes were judged to be dead if they did not respond to stimulus using a small, metal wire. Ten replicates were performed, and three hundred of nematodes were examined for each replicate. We used the body length to assess growth of nematodes. The body length was determined by measuring the flat surface area of nematodes using the Image-Pro® Express software. Ten replicates were performed. We used the brood size to evaluate reproduction of nematodes. To assay the brood size, number of the offspring at all stage beyond the egg was counted. Ten replicates were performed. To assay the pumping rate, nematodes were placed onto NGM plates with food, and left undisturbed for 1-hr before measuring. Pharyngeal pumping was counted for 1 min under DIC optics with a Ziess axioscope. To assay the mean defecation cycle length, individual animal was examined for a fixed number of cycles, and a cycle period was defined as the interval between the initiations of two successive posterior body-wall muscle contraction steps. Thirty replicates were performed. We used the head thrash, and body bend to evaluate locomotion behavior of nematodes [61]. To assay the head thrash, every examined nematode was transferred into a microtiter well containing 60 µL of K medium on the top of agar without food, and head thrashes were counted for 1-min after a 1-min recovery period. A thrash was defined as a change in the direction of bending at the mid body. To assay the body bend, nematodes were picked onto a second plate without food and scored for the number of body bends in an interval of 20 sec. A body bend was counted as a change in the direction of the part of nematodes corresponding to the posterior bulb of the pharynx along the *y* axis, assuming that the nematode was traveling along the *x* axis. Fifty replicates were examined per treatment.

### Intestinal autofluorescence

Intestinal autoflorescence caused by lysosomal deposits of lipofuscin can accumulate over time in aging nematodes [62]. Images were collected for endogenous intestine fluorescence using a 525-nm bandpass filter and without automatic gain control in order to preserve the relative intensity of different animal's fluorescence. We used the Magnafire® software (Olympus, Irving, TX, USA) to analyze the fluorescence of color images taken for the documentation of results. Lipofuscin levels were measured using ImageJ Software (NIH Image) by determining average pixel intensity in each animal's intestine. Twenty nematodes for each treatment were counted.

### ROS production

To quantify whether Fe_2_O_3_-NPs treatment activated the oxidative damage, the ROS production was assayed. The examined nematodes were transferred to M9 buffer containing 1 µM CM-H2DCFDA to pre-incubate for 3-h at 20°C, and then mounted on agar pads for the examination with a laser scanning confocal microscope (Leica, TCS SP2, Bensheim, Germany) at 488 nm of excitation wavelength and 510 nm of emission filter. Relative fluorescent intensities of the intestine were semi-quantified. The semiquantified ROS was expressed as relative fluorescent units (RFU). Twenty nematodes for each treatment were counted.

### Statistical analysis

All data were expressed as means ± standard error of the mean (S.E.M.). Statistical analysis was performed using SPSS 12.0 (SPSS Inc., Chicago, IL, USA). Analysis of variance (ANOVA) was used to determine the significance of differences between the groups. Probability levels of 0.05 and 0.01 were considered statistically significant. Associations of ROS production with lethality, growth, reproduction, locomotion behavior, pharyngeal pumping, defecation, and intestinal autolfuorescence were assessed with linear regression analysis.

## Supporting Information

Figure S1 **Effects of double mutations of** ***sod-2*** **and** ***sod-3*** **genes on locomotion behavior and ROS production in nematodes exposed to 10 μg/L of DMSA coated Fe_2_O_3_-nanoparticles from L1-larvae to day-8 adult.** (A) Effects of double mutations of *sod-2* and *sod-3* genes on head thrash in nematodes exposed to 10 μg/L of DMSA coated Fe_2_O_3_-nanoparticles from L1-larvae to day-8 adult. (B) Effects of double mutations of *sod-2* and *sod-3* genes on body bend in nematodes exposed to 10 μg/L of DMSA coated Fe_2_O_3_-nanoparticles from L1-larvae to day-8 adult. (C) Effects of double mutations of *sod-2* and *sod-3* genes on ROS production in nematodes exposed to 10 μg/L of DMSA coated Fe_2_O_3_-nanoparticles from L1-larvae to day-8 adult. Bars represent mean ±S.E.M. **p<0.01.
(DOC)

Table S1 **Associations of ROS production with lethality, growth, reproduction, locomotion behavior, metabolism, and intestinal autofluorescence in nematodes exposed to DMSA coated Fe_2_O_3_-NPs as assayed by linear regression analysis.**
(DOC)

Table S2 **Physicochemical properties of Fe_2_O_3_-nanoparticles.**
(DOC)
