# Supplementary material for: Evaluation of Environmental Safety Concentrations of DMSA Coated Fe2O3-NPs Using Different Assay Systems in Nematode Caenorhabditis elegans
Source: PLoS One. 2012 Aug 17;7(8):e43729. doi: 10.1371/journal.pone.0043729 (PMC3422352; doi:10.1371/journal.pone.0043729)
Supplement: Figure S1 — Effects of double mutations of sod-2 and sod-3 genes on locomotion behavior and ROS production in nematodes exposed to 10 μg/L of DMSA coated Fe2O3-nanoparticles from L1-larvae to day-8 adult. (A) Effects of double mutations of sod-2 and sod-3 genes on head thrash in nematodes exposed to 10 μg/L of DMSA coated Fe2O3-nanoparticles from L1-larvae to day-8 adult. (B) Effects of double mutations of sod-2 and sod-3 genes on body bend in nematodes exposed to 10 μg/L of DMSA coated Fe2O3-nanoparticles from L1-larvae to day-8 adult. (C) Effects of double mutations of sod-2 and sod-3 genes on ROS production in nematodes exposed to 10 μg/L of DMSA coated Fe2O3-nanoparticles from L1-larvae to day-8 adult. Bars represent mean ±S.E.M. **p<0.01. (DOC) [file pone.0043729.s001.doc]

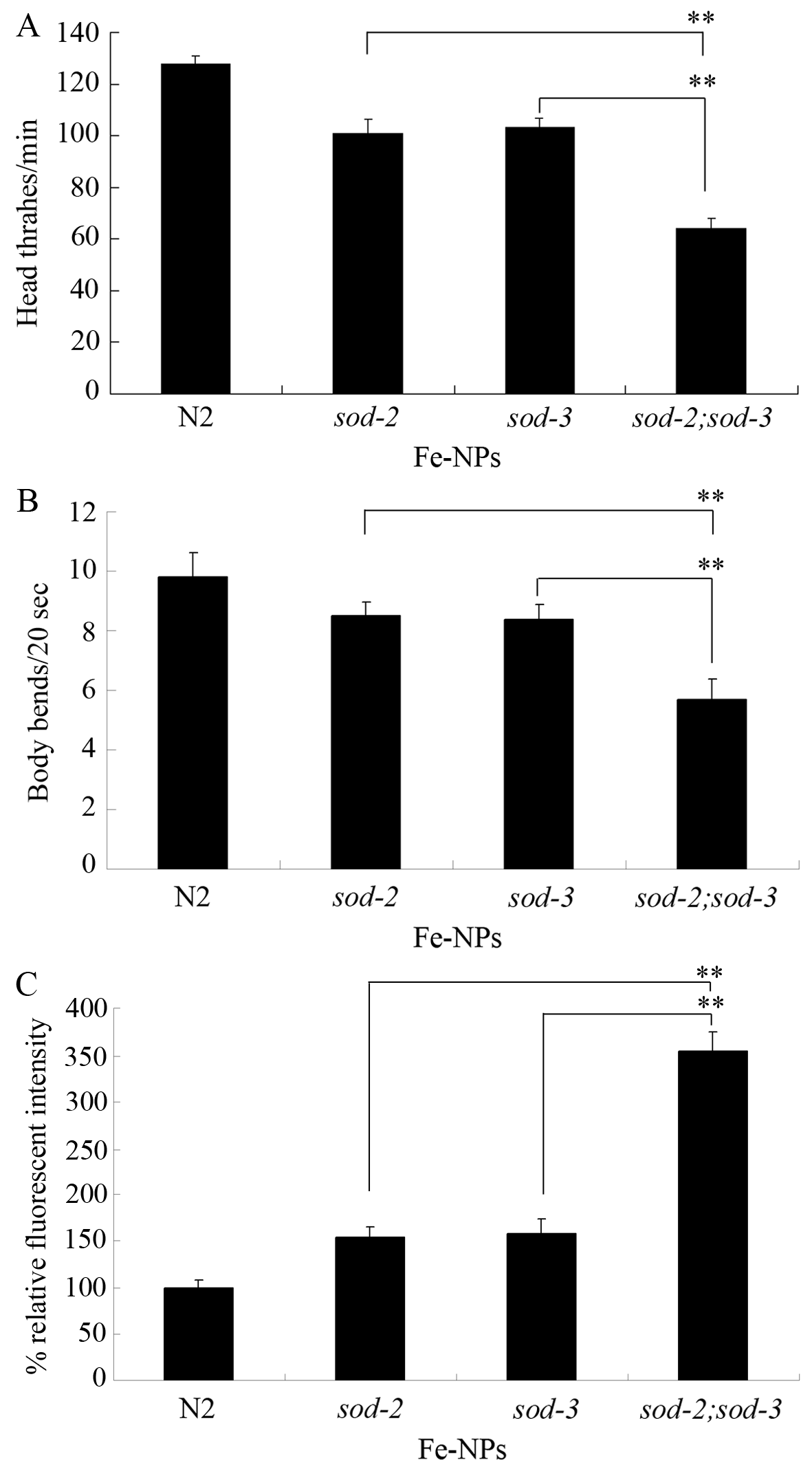


**Figure S1. Effects of double mutations of *sod-2* and *sod-3* genes on locomotion behavior and ROS production in nematodes exposed to 10 μg/L of DMSA coated Fe_2_O_3_-nanoparticles from L1-larvae to day-8 adult.** (A) Effects of double mutations of *sod-2* and *sod-3* genes on head thrash in nematodes exposed to 10 μg/L of DMSA coated Fe_2_O_3_-nanoparticles from L1-larvae to day-8 adult. (B) Effects of double mutations of *sod-2* and *sod-3* genes on body bend in nematodes exposed to 10 μg/L of DMSA coated Fe_2_O_3_-nanoparticles from L1-larvae to day-8 adult. (C) Effects of double mutations of *sod-2* and *sod-3* genes on ROS production in nematodes exposed to 10 μg/L of DMSA coated Fe_2_O_3_-nanoparticles from L1-larvae to day-8 adult. Bars represent mean ± S.E.M. ***p* < 0.01.
