# Supplementary material for: Evaluation of Environmental Safety Concentrations of DMSA Coated Fe2O3-NPs Using Different Assay Systems in Nematode Caenorhabditis elegans
Source: PLoS One. 2012 Aug 17;7(8):e43729. doi: 10.1371/journal.pone.0043729 (PMC3422352; doi:10.1371/journal.pone.0043729)
Supplement: Table S1 — Associations of ROS production with lethality, growth, reproduction, locomotion behavior, metabolism, and intestinal autofluorescence in nematodes exposed to DMSA coated Fe2O3-NPs as assayed by linear regression analysis. (DOC) [file pone.0043729.s002.doc]

**Table S1. Associations of ROS production with lethality, growth, reproduction, locomotion behavior, metabolism, and intestinal autofluorescence in nematodes exposed to DMSA coated Fe_2_O_3_-NPs as assayed by linear regression analysis.**

| Dependent variable | Independent variable | | | | | |
| --- | --- | --- | --- | --- | --- | --- |
|  | ROS production | | | | | |
|  | L4 larvae (24-hr) | | L1-larvae  to adult | | L1-larvae  to day-8 adult | |
|  | *R^2^* | *P* value | *R^2^* | *P* value | *R^2^* | *P* value |
| Lethality | - | - | - | - | 0.920 | < 0.01 |
| Body bend | 0.730 | < 0.05 | 0.739 | < 0.05 | 0.947 | < 0.01 |
| Head thrash | 0.795 | < 0.05 | 0.842 | < 0.05 | 0.920 | < 0.01 |
| Body length | 0.856 | < 0.01 | 0.688 | < 0.05 | 0.883 | < 0.01 |
| Brood size | 0.913 | < 0.01 | 0.881 | < 0.01 | - | - |
| Pumping rate | 0.861 | < 0.01 | 0.863 | < 0.01 | 0.828 | < 0.05 |
| Mean defecation cycle length | 0.954 | < 0.01 | 0.884 | < 0.01 | 0.842 | < 0.05 |
| Intestinal autofluorescence | 0.977 | < 0.01 | 0.993 | < 0.01 | 0.781 | < 0.05 |
