# Supplementary material for: Evaluation of Environmental Safety Concentrations of DMSA Coated Fe2O3-NPs Using Different Assay Systems in Nematode Caenorhabditis elegans
Source: PLoS One. 2012 Aug 17;7(8):e43729. doi: 10.1371/journal.pone.0043729 (PMC3422352; doi:10.1371/journal.pone.0043729)
Supplement: Table S2 — Physicochemical properties of Fe2O3-nanoparticles. (DOC) [file pone.0043729.s003.doc]

**Table S2. Physicochemical properties of Fe_2_O_3_-nanoparticles.**

| Particle size | 9 ± 1 nm  (TEM measurement) |
| --- | --- |
| Crystal structure | Inverse cubic spinel structure |
| Zeta potential | -69 ± 3 mV |
| Hydrodynamic mean diameter | 85 ± 5 nm (DLS measurement) |
| Specific surface area | 89 ± 3 m^2^/g |
